# The association between insulin sensitivity indices, ECG findings and mortality: a 40-year cohort study

**DOI:** 10.1186/s12933-021-01284-9

**Published:** 2021-05-06

**Authors:** Yonatan Moshkovits, David Rott, Angela Chetrit, Rachel Dankner

**Affiliations:** 1 Department of Epidemiology and Preventive Medicine, School of Public Health, Sackler School of Medicine, Tel Aviv University, Tel Aviv, Israel; 2 Leviev Heart Center, Sheba Medical Center, Ramat Gan, Israel; 3 Unit for Cardiovascular Epidemiology, The Gertner Institute for Epidemiology and Health Policy Research, Ramat Gan, Israel

**Keywords:** Diabetes mellitus, Insulin sensitivity indices, ECG findings, All-cause mortality, Cardiovascular mortality

## Abstract

**Background:**

Type 2 Diabetes is a major risk factor for cardiovascular (CV) mortality. Insulin resistance can be evaluated non-invasively by insulin sensitivity indices (ISI) such as the Mcauley index (MCAi), which is a function of the fasting insulin and triglycerides. Currently, the association between ISIs and ECG findings and all-cause and CV mortality is still not established in a large scale and heterogeneous population.

**Method:**

In a prospective study of the Israel cohort on Glucose Intolerance, Obesity and Hypertension (GOH) second phase (1979–1982) 1830 men and women were followed until December-2016 for CV-mortality and December-2019 for all-cause mortality. ECGs were recorded and OGTTs performed during baseline. ISIs were categorized into quartiles and evaluated against ECG findings and all-cause and CV-mortality.

**Results:**

Mean age at baseline was 52.0 ± 8.1 years, and 75 (15.2%) and 47 (25.3%) participants in the upper quartiles (Q_2-4_) and the lower quartile (Q_1_) of the MCAi, presented with Ischemic changes on ECG respectively (*p* = 0.02). Multivariable analysis showed higher odds for ECG ischemic changes, for individuals in Q_1_-MCAi (adjusted-OR = 1.7, 95% CI 1.02–2.8), compared with Q_2-4_-MCAi, which attenuated when excluding individuals with diabetes (adjusted-OR = 1.6, 95% CI 0.9–2.7, *p* = 0.09).

Median follow up for all-cause and for cardiovascular mortality was 31 years and 37 years, respectively. Cox proportional-hazards regression showed an increased risk for all-cause mortality for individuals in Q_1_-MCAi (HR = 1.2, 95% CI 1.02–1.3) as well as an increased risk for CV-mortality (HR = 1.4, 95%CI 1.1–1.8) compared with Q_2-4_-MCAi. Individuals in Q_4_-Ln Homeostatic model assessment- Insulin Resistance (HOMA-IR) and Q_1_- Quantitative Insulin Sensitivity Check Index (QUICKI) also presented with increased risk for all-cause-mortality (HR = 1.2, 95%CI 1.04–1.4; and HR = 1.2, 95% CI 1.04–1.4, respectively). Other ISIs did not show significant associations with CV-mortality.

**Conclusion:**

Higher insulin-resistance, according to the MCAi, associated with ECG-changes, and with greater risk for all-cause and CV-mortality over a 40-year follow-up. The MCAi may be considered as an early predictive and prognostic biomarker for CV-morbidity and mortality in adults.

**Supplementary Information:**

The online version contains supplementary material available at 10.1186/s12933-021-01284-9.

## Introduction

Type 2 diabetes mellitus is one of the most common chronic diseases of the modern world. According to the American diabetes association [1], the prevalence of diabetes in the general population in 2015 was 9.4% (30.3 million Americans) and 25.2% in the elderly population. In addition, 1.5 million new cases are reported every year [1, 2].

Diabetes is a well-known risk factor for micro and macro-vascular complications [3–5] such as myocardial infarction, cerebral vascular accident, retinopathy and nephropathy. Recent studies have shown that even prediabetes was associated with vascular complications [5, 7]. The underlying pathophysiological mechanisms of the disease are depleted pancreatic beta cell function and systemic insulin resistance (IR).

In order to evaluate pancreatic beta cells function and IR in a non-invasive manner, compared with the gold standard and intrusive euglycemic insulin clamp, a number of indices were developed and validated based on insulin and glucose blood levels [8–12]. Commonly used indices include the Homeostatic model assessment (HOMA) [8, 9], the Matsuda Insulin Sensitivity Index (MISI) [10], the Quantitative Insulin Sensitivity Check Index (QUICKI) [11] and the Mcauley index (MCAi) [12]. Selected insulin sensitivity indices description, normal values, formulas and classification are detailed in Table 1.

**Table 1:** Insulin sensitivity indices description, reported cut points for normoglycemia, and insulin resistance classification in the present study

Index	Formula	Description	Normoglycemia cut points	IR Classification and calculation in the present study
HOMA-IR	$\mathrm{FPI }\times \frac{\mathrm{FPG }}{405}$	Linear model that evaluates insulin resistance Reflects the interaction between insulin secretion and hepatic glucose production Based on fasting glucose and insulin plasma levels. [8, 9, 13]	< 2–2.5	HOMA-IR increasing values depicts insulin resistance [13] (13) HOMA-IR was not normally distributed and was logarithmically transformed implementing natural log (Ln) on the equation Upper quartile (> 1.6) of Ln HOMA-IR (Q_4_) was compared to the lower quartiles (< 1.6- Q_1-3_)
HOMA-%B	$360\times \frac{\mathrm{FPI}}{(\mathrm{FPG }-63)}$	Linear model that evaluates pancreatic beta cell function Reflects the interaction between insulin secretion and hepatic glucose production Based on fasting glucose and insulin plasma levels. [8, 9, 13]	> 100% Depend on the population [38, 39]	HOMA-%B decreasing values depicts beta cells dysfunction (13) HOMA-%B was not normally distributed and was logarithmically transformed implementing natural log (Ln) on the equation Ln HOMA-%B lower quartile (< 4.5- Q_1_) was compared with upper Ln HOMA-%B quartiles (> 4.5- Q_2-4_)
MISI	$\frac{\mathrm{10,000}}{\sqrt{\left(\mathrm{FPG}\times \mathrm{FPI}\right)\times [\mathrm{mean glu x mean ins}]}}$	Evaluate the total body insulin resistance levels Based on fasting glucose and insulin plasma levels and OGTT [10]	> 4.3	MISI decreasing values depicts insulin resistance (IR) MISI mean glucose plasma levels were calculated using: fasting glucose, 60 and 120 min glucose after an oral administration of 100gr glucose. Mean insulin plasma levels were calculated using fasting insulin and insulin levels at 30, 60 and 120 min after the oral administration of 100gr glucose MISI was not normally distributed and was logarithmically transformed implementing natural log (Ln) on the equation Ln MISI lower quartile (< 0.89-Q_1_) was compared with upper Ln MISI quartiles (> 0.89-Q_2-4_)
QUICKI	$\frac{1}{\mathrm{Log FPI}+\mathrm{Log FPG}}$	Equivalent to the HOMA model and considered as log HOMA-IR Based on fasting glucose and insulin plasma levels Correlate better than HOMA with SI Clamp [11]	> 0.38 in non-obese	QUICKI decreasing values depicts insulin resistance. Lower quartile (< 0.3-Q_1_) of QUICKI was compared to upper QUICKI quartiles (> 0.3-Q_2-4_)
MCAi	${e}^{\left[2.63-0.28*Ln \mathrm{FPI}-0.31*Ln trig\right]}$	Provide a simple screening tool for the assessment of insulin resistance in the population. Based on fasting insulin and triglycerides plasma levels (12)	< 5.8; ≤ 0.63 in diabetes [44]	MCAi decreasing values depicts insulin resistance. MCAi lower quartile (< 3.2-Q_1_) was compared to upper MCAi quartiles (> 3.2-Q_2-4_)

*HOMA-IR* Homeostatic model assessment -Insulin resistance, *FPI* fasting insulin levels in $\left[\frac{\mathrm{mU}}{\mathrm{L}}\right]$, *FPG* fasting glucose levels in $[\frac{\mathrm{mg}}{\mathrm{dl}}]$, *HOMA-%B* Homeostatic model assessment—percent beta cell function (B), *MISI* Matsuda Insulin Sensitivity Index, *Mean Glu* mean glucose during OGTT, *Mean Ins* mean insulin during oral glucose tolerance test (OGTT); *QUICKI* Quantitative Insulin Sensitivity Check Index; *MCAi* Mcauley index, Trig, fasting triglycerides levels in $\left[\frac{\mathrm{mMole}}{\mathrm{L}}\right]$

The association between abnormal ECG findings and cardiovascular mortality and morbidity was previously described [14, 15].

Previous studies have examined the association between metabolic syndrome and insulin sensitivity indices with ECG abnormalities, with inconclusive results [15–22]. Bhatt et al. showed an association between log MISI and HOMA-derived measures with pathologic Q waves and left ventricular hypertrophy (LVH) on ECGs, on a homogeneous sample of 1671 type 2 diabetic patients [22].

The association between abnormal values of insulin sensitivity indices and all cause and cardiovascular mortality is still not well established and results are contradicting [23, 24]. Barr et al. [23] did not demonstrate a significant association with abnormal HOMA of insulin sensitivity (HOMA-%S) quintiles and all-cause mortality (quintile Hazard ratio 1.1, 95% CI: 0.8–1.7) on 8533 subjects aged > 35 years as part of the population-based Australian Diabetes, Obesity and Lifestyle study. Nevertheless De Boer et al. [24] showed on a population of 3138 older adults (age ≥ 65 years) without diabetes and after a 14.7 year median follow-up, an increased risk for all-cause mortality for individuals in the lower quartile of MISI (Hazard ratio 1.23, 95%CI: 1.11–1.44). However, after adjustment for eGFR the association was no longer statistically significant.

The discovery of innovative predictive and prognostic factors in the general population and specifically in patients with diabetes and those with prediabetes, beyond the conventional risk factors, is crucial in the prevention and reduction of cardiovascular morbidity and mortality.

We evaluated the association between insulin sensitivity indices and ECG findings, and their association to all-cause and cardiovascular mortality over a 40-year follow-up.

## Methods

### Study design and population

The study is based on the cohort of the Israel Glucose Intolerance, Obesity and Hypertension (GOH) study [25]. This is a prospective study, which began in 1967 and included 8400 Israeli Jews that were randomly sampled from the Israel population registry, according to sex (50% of each sex), birth decade (1912–1921; 1922–1931; 1932–1941) and ethnic origin (Yemenite, Asian, North Africans, and European-North Americans) stratification. Initially, the study aimed to determine the prevalence of hypertension in the Israeli population. During the second phase of the study (1979–1982), subjects were invited to regional medical centers, and during a single visit underwent medical interviews, anthropometric measurements, extensive blood tests after a 12 h fast, including glucose and insulin levels during fasting and during a 2 h oral glucose tolerance test (OGTT) and resting ECG recording [26, 27].

Inclusion criteria for the present study were the presence of a resting ECG, as well as data on fasting glucose and fasting insulin plasma levels.

Out of 3726 participants, primarily interviewed during the second phase, 2469 underwent resting ECG recording, 2802 were tested for fasting glucose levels and 1843 were tested for fasting insulin levels. When comparing the distribution of age, sex, and ethnic origin between those with ECG, which is the first inclusion criteria, to those with the full set of data, no difference were noted.

Blood tests were evaluated using a single lab. Plasma glucose was determined with the automated Technicon Autoanalyser II (Technicon Instruments Corp, Tarrytown, NY) with the use of potassium ferricyanide reduction; plasma insulin level was determined in duplicate with the Phadebas Radioimmunoassay kit (Pharmacia Diagnostics Inc. Piscataway, NJ). The methodology of the GOH study was described in details by Dankner et al. [25].

Participants were followed until December 2019 for all-cause mortality and until December 2016 for cause specific mortality. Information on vital state and date of death were obtained from the National Population Registry, which is continuously updated, and causes of death, coded and available for research purposes within a 2–3 years lag, from the Israel Ministry of Health.

Cohort members agreed to participate in the study, and the study protocol was approved by the 1975 Declaration of Helsinki as reflected in a priori approval by the Sheba Medical Center's IRB.

### Glycemic status

Diabetes and prediabetes were defined according to ADA's glucose cut point criteria. [28] Prediabetes was defined as fasting plasma glucose 5.55–6.94 mmol/L (100–125 mg/dl) or 2 h post OGTT plasma glucose 7.77–10.54 mmol/L (140–199 mg/dl) and no reporting of having diabetes or using anti-diabetes medications; Diabetes was defined as fasting plasma glucose > 6.94 mmol/L (125 mg/dl) or 2 h post OGTT plasma glucose > 10.54 mmol/L (199 mg/dl) or reporting of having diabetes or using anti-diabetes medications.

### ECG findings

Twelve-lead ECG recordings were interpreted and encoded by a single cardiologist according to the Minnesota code classification system [26]. ECG findings were classified into subgroups using the Minnesota code manual 2009 and based on findings from a previous publication on the study population [30] into (Additional file 1: Table S2): Arrhythmia, Right axis deviation, Left axis deviation, Atrioventricular conduction defect, Ventricular conduction defect, ST Junction (J) and segment depression, Miscellaneous findings, Nonspecific T wave changes, Nonspecific ST changes, Ischemic changes, Left Axis Deviation + Nonspecific T wave changes.

### Insulin sensitivity indices (ISI)

Commonly used insulin sensitivity indices, examined in the current study, included the HOMA-Insulin resistance (IR) HOMA-IR, and the beta cell function HOMA-%B [8, 9], the MISI [10], the QUICKI [11] and the MCAi [12]. Indices were calculated and analyzed according to quartiles, a common and extensively described method for the classification and establishment of ISIs cutoffs in the literature [24], as described in Table 1.

### Endpoints

The primary outcome was 40-year all-cause mortality. The secondary outcome was cardiovascular mortality. Individual follow-up time was calculated starting at the examination date (physical examination and blood tests) during the second phase and until time of death or end of follow up- earliest of these. Primary cause of death was reported using International Classification of Diseases (ICD) 9 or ICD 10.

The Sheba Medical Center Review Board provided approval for this study (approval number 1180). All patients gave their verbal consent to participate in the study during baseline data collection.

### Statistical methods

In-group differences between ECG findings were evaluated using the Chi square test or the Fisher’s exact test for small cells and the Student t test for normally distributed variables or the Mann–Whitney test for nonparametric variables, with the two-sided p-values (p) set at the 0.05 level of significance. The association between insulin sensitivity indices and ECG findings was evaluated using a multivariable logistic regression model and presented by Odd Ratios with 95% confidence interval (95%CI) adjusted for age, sex, ethnicity, smoking status, BMI, blood pressure, cholesterol and glycemic state. Fasting glucose models were not adjusted for glycemic state due to multi-collinearity.

The associations between insulin sensitivity indices and 40-year all cause and CVD mortality were evaluated using Cox proportional hazards models adjusted for the same covariates as mentioned above. ISI were tested in a separate model each. Proportional hazards assumptions were tested in the models by entering into the model an interaction term between time-to-event for each covariate and by log minus log plot. A test for multi-collinearity was performed using Spearman's rank correlation coefficient for model covariates. Covariates with a correlation above 60% were not included in the same model. Kaplan Meier survival curves for ECG findings and insulin sensitivity indices were compared using log-rank test. Statistical analysis was performed using SPSS version 23.0.

## Results

### Baseline characteristics (Table 2)

**Table 2:** Cohort baseline characteristics according to ECG findings

		ECG		
Baseline characteristic	Total, n (%)	Normal, n (%)	Abnormal, n (%)	*P*-value
	N = 1830	n = 915	n = 915	
Age (years), mean ± SD	52.0 ± 8.1	50.3 ± 7.8	53.7 ± 7.9	< 0.001
Sex				0.092
Male	936 (51.1)	450 (49.2)	486 (53.1)	
Female	894 (48.9)	465 (50.8)	429 (46.9)	
Origin				0.334
Middle East	474 (25.9)	241 (26.3)	233 (25.5)	
North Africa	336 (25.9)	161 (17.6)	175 (19.1)	
Yemen	401 (21.9)	189 (26.3)	212 (23.2)	
Europe-America	619 (33.8)	324 (35.4)	295 (32.2)	
Smoking status ^a^				0.939
Ever smoked	728 (39.8)	365 (39.9)	363 (39.7)	
Never-Smoker	1101 (60.2)	550 (60.1)	551 (60.3)	
Glycemic state				< 0.001
Normoglycemia	741 (40.5)	422 (46.1)	319 (34.9)	
Prediabetes	871 (47.6)	406 (44.4)	465 (50.0)	
Diabetes	218 (11.9)	87 (9.5)	131 (14.3)	
Blood Pressure (mmHg), mean ± SD				
Systolic	132.5 ± 26.5	128.5 ± 23.1	136.6 ± 28.9	< 0.001
Diastolic	84.0 ± 14.9	82.6 ± 13.6	85.4 ± 16.0	< 0.001
BMI (${\mathrm{Kg}/\mathrm{m}}^{2}$) ^b^, median [IQR]	25.5 [5.1]	25.3 [4.7]	25.7 [5.2]	0.011
Normal	807 (44.1)	431 (47.1)	376 (41.1)	0.007
Overweight	738 (40.3)	362 (39.6)	376 (41.1)	
Obese	285 (15.6)	122 (13.3)	163 (17.8)	
Fasting glucose (mg/dl)	105.5 ± 29.8	103.6 ± 28.6	107.4 ± 30.9	< 0.001
Q_1-3_	1348 (73.7)	711 (77.7)	637 (69.6)	< 0.001
Q_4_	482 (26.3)	204 (22.3)	278 (30.4)	
Fasting insulin (mU/L)	15.9 ± 11.9	15.9 ± 11.9	16.8 ± 11.8	0.014
Q_1-3_	697 (76.2)	697 (76.2)	668 (73)	0.12
Q_4_	465 (25.4)	218 (23.8)	247 (27)	
Total cholesterol ^c^ (mg/dl), mean ± SD	221.1 ± 54.4	217.9 ± 53.9	224.30 ± 54.9	0.012
Normal	595 (32.5)	320 (35.0)	275 (30.1)	0.058
Borderline-high	573 (31.3)	269 (29.4)	304 (33.2)	
High	662 (36.2)	326 (35.6)	336 (36.7)	
Triglycerides (mg/dl), median [IQR]	110 [75]	110 [75]	115 [75]	0.02
MISI, median [IQR]	3.5 [2.6]	3.7 [2.3]	23.2 [2.2]	< 0.001
Ln MISI, mean ± SD	1. 2 ± 0.5	1.3 ± 0.5	1.2 ± 0.6	< 0.001
Q_1_	267 (25)	116 (21.2)	151 (28.8)	0.004
Q_2-4_	803 (75)	430 (78.8)	373 (71.2)	
HOMA-IR,Median [IQR]	3.4 [2.4]	3.2 [2.3]	3.6 [2.7]	0.001
Ln HOMA-IR, mean ± SD	1.3 ± 0.6	1.2 ± 0.6	1.3 ± 0.7	0.004
Q_1-3_	1373 (75)	718 (78.5)	655 (71.6)	0.001
Q_4_	457 (25)	197 (21.5)	260 (28.4)	
HOMA-%B, Median [IQR]	134.7 [100.2]	135.2 [97.1]	134.1 [104.9]	0.24
Ln HOMA-%B, mean ± SD	4.9 ± 0.6	4.9 ± 0.7	4.88 ± 0.6	0.44
Q_1_	456 (25)	211 (23.1)	245 (26.9)	0.06
Q_2-4_	1370 (75)	703 (76.9)	667 (73.1)	
QUICKI, mean ± SD	0.319 ± 0.03	0.321 ± 0.03	0.318 ± 0.03	0.011
Q_1_	457 (25)	197 (21.5)	260 (28.4)	0.001
Q_2-4_	1373 (75)	718 (78.5)	655 (71.6)	
MCAi, mean ± SD	3.9 ± 0.96	3.9 ± 0.95	3.8 ± 0.97	0.007
Q_1_	448 (25)	208 (23.1)	240 (26.9)	0.06
Q_2-4_	1347 (75)	694 (76.9)	653 (73.1)	

*HOMA-IR* Homeostatic model assessment -Insulin resistance, *HOMA-%B* Homeostatic model assessment—percent beta cell function; MISI, Matsuda Insulin Sensitivity Index, *QUICKI* Quantitative Insulin Sensitivity Check Index, *MCAi* Mcauley index

^a^Smoking status classification: Smoker-currently or past smoker. Nonsmoker-never smoked; ^b^BMI categories: Normal < 25 kg/m^2^, Overweight, 25–29.9 kg/m^2^, Obese- BMI ≥ 30 kg/m^2^; ^c^Total cholesterol classification: Normal < 200 mg/dl, Borderline-high, 200–239 mg/dl, High ≥ 240 mg/dl

The final cohort comprised of 1830 subjects who met the inclusion criteria, of whom 915 (50%) examinees had ECG findings that were classified as abnormal. Mean age of individuals with abnormal ECG was 53.7 ± 7.9 years whereas that of individuals with normal ECG was 50.3 ± 7.8 years (P < 0.001), with a greater male proportion in the abnormal vs the normal ECG group, 53.1 and 49.2% respectively, p = 0.09. Blood pressure, BMI, total cholesterol, fasting triglycerides, fasting glucose and fasting insulin, as well as diabetes were significantly higher in the abnormal ECG group. No differences were observed between the two ECG groups regarding ethnic origin and smoking status. When categorized, all 5 ISIs indicated a greater insulin resistance in the ECG abnormal group. In MISI, 28.8% and 21.2% individuals belonged to the Q1 in the abnormal and the normal ECG groups, respectively, p = 0.004. The respective proportions for the MCAi were 26.9% and 23.1%, p = 0.06.

### ECG findings

Ischemic changes, defined as Q and QS abnormal patterns or ST segment elevation, were observed in 128 (7%) participants (Additional file 1: Table S2).

**Fig. 1:**
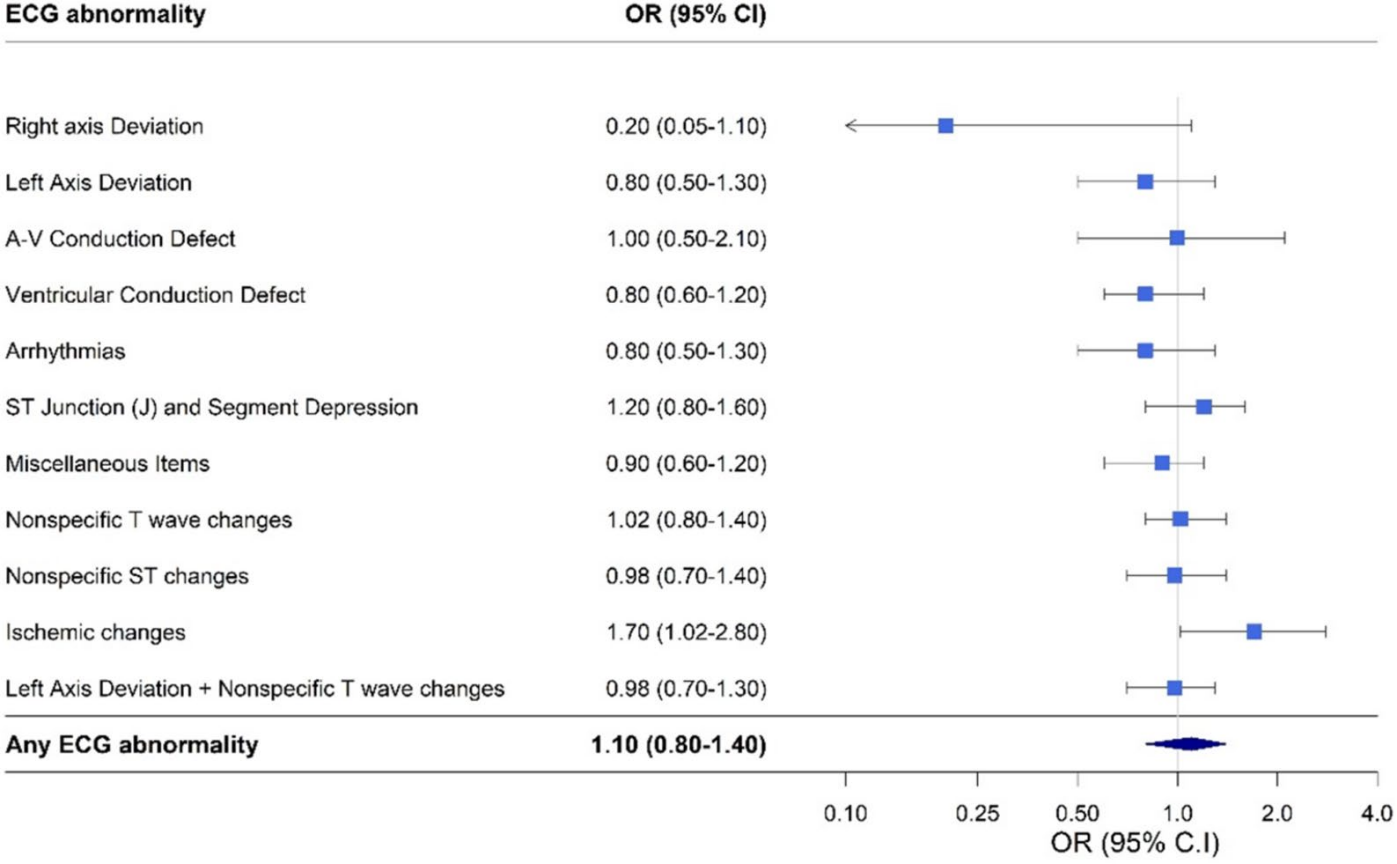
Odds Ratios for the association between higher insulin resistance, according to the Mcauley index (MCAi) lower quartile (Q_1_) compared with upper quartiles (Q_2-4_), and ECG findings. Multivariable ^a^ logistic regression analysis. ^a^Adjusted for: Age, Sex, Origin, BMI category, Smoking category, Systolic Blood Pressure, Glycemic state, Total Cholesterol. BMI categories: Normal < 25 kg/m^2^, Overweight, 25–29.9 kg/m^2^, Obese, BMI ≥ 30 kg/m^2^; Smoking categories: current or past smoker vs never smoked; Glycemic state: normoglycemia, prediabetes, diabetes. Total cholesterol classification: Normal < 200 mg/dl, Borderline-high, 200–239 mg/dl, High ≥ 240 mg/dl

All ISIs were significantly associated with "any ECG" abnormality, although after adjustment for age, sex, origin, BMI, blood pressure, cholesterol, smoking and glycemic state, none of the ISI remained statistically significant. Ischemic changes on ECG were associated with greater adjusted odds for the MCAi Q_1_ compared to MCAi Q_2-4_ (OR = 1.7, 95%CI: 1.02–2.8, P = 0.04) (Fig. 1, Additional file 1: Table S3). Male sex (OR = 3.1, 95%CI: 1.9–5.2, *P* < 0.001), older age (OR = 1.1, 95%CI: 1.07–1.1, *P* < 0.001) obesity (OR = 2.1, 95%CI: 1.1–4.2, *P* = 0.03) and higher blood pressure (OR = 1.01, 95%CI: 1.0–1.02, *P* = 0.03) were also associated with increased risk for ischemic changes on ECG, whereas increased triglycerides did not show an association (OR = 1.0, 95%CI: 0.99–1.0, *P* = 0.7). The association of the MCAi with the various ECG abnormalities are presented in Fig. 1, showing an overall odd for any ECG abnormality of 1.10 (95% CI: 0.80–1.40).

In the diabetes group, a large proportion of individuals (47%-64%) belonged to the abnormal quartiles of the ISIs as expected. In the prediabetes group, about 27–30% were categorized in the abnormal ISIs (Additional file 1: Table S4a).

No statistically significant associations between the other ISIs and ECG findings were observed in the adjusted multivariable analyses (Additional file 1: Table S3). A sensitivity analysis excluding diabetic individuals from the models is presented in Additional file 1: Table S4b, showing borderline association between the MCAi and ischemic changes (adjusted OR = 1.6, 95% CI 0.9–2.7, *p* = 0.09) for Q_1_ vs Q_2-4_.

### All-cause and cause specific mortality

Participants were followed until December 2019 for all-cause mortality and until December 2016 for cardiovascular mortality. Median follow up was 31 years and 1276 (69.7%) of all participants died during that period. Median follow up for cardiovascular mortality was 37 years and 377 (20.6%) participants died from cardiovascular causes. Additional file 1: Table S1 is presenting the baseline characteristics of the study cohort according to vital status and cause of death. As expected, those who died from all cause and from cardiovascular mortality were older than those who remained alive by the end of the follow-up. Those who died were of male predominance, had a higher proportion of diabetes, were more hypertensive, and obese. Mean fasting glucose values were in the prediabetic range in those who died from all-cause and from cardiovascular causes compared to those remaining alive (109 ± 34 and 115 ± 43 vs. 97 ± 14 mg/dl, respectively), and their total cholesterol was higher as well (224 ± 55 and 232 ± 57 vs 214 ± 52 mg/dl, respectively). Insulin resistance was more pronounced in those who died than those remaining alive, as evident by all 5 IRIs.

**Fig. 2:**
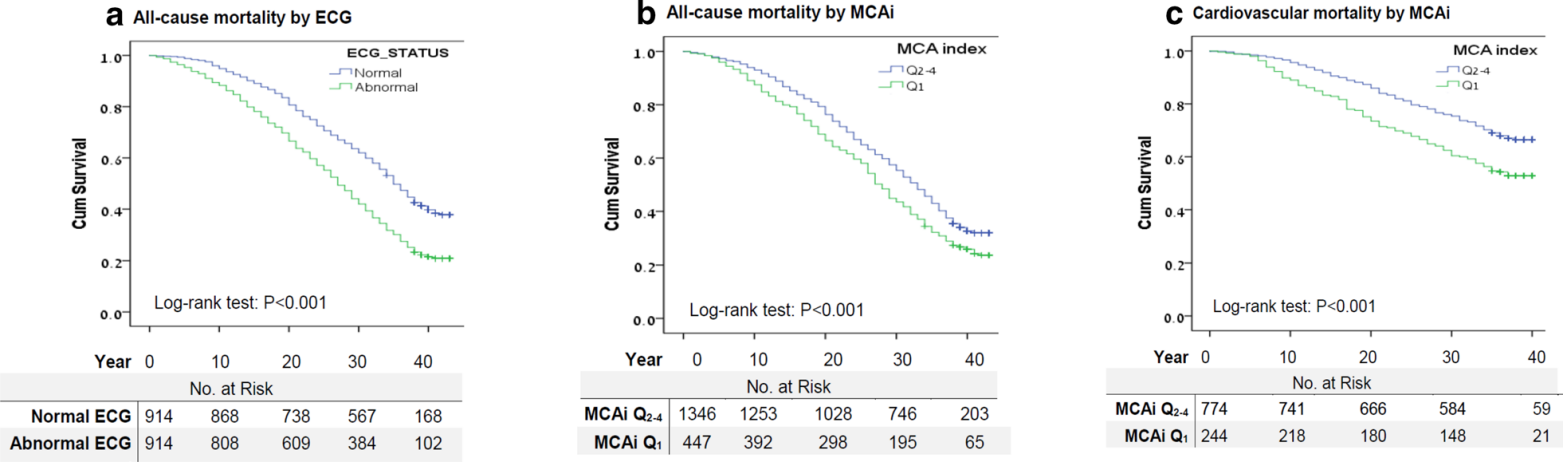
Kaplan–Meier survival curves for **a** any ECG abnormality and all-cause mortality; **b** Insulin resistance according to the Mcauley index (MCAi) Q_1_ vs Q_2-4_ and all-cause mortality; and **c** Insulin resistance according to the Mcauley index (MCAi) Q_1_ vs Q_2-4_ and cardiovascular mortality. Median survival in the normal ECG group was 35 years (95% CI, 33.8–36.2) and 27 years (95% CI, 25.8–28.2) in the abnormal ECG group. Median survival in the lower MCAi quartile (Q_1_) was 28 (95% CI, 26.6–29.4) years and 33 (95% CI, 31.9–34.1) years in the upper MCAi quartiles (Q_2-4_). Mean survival for cardiovascular mortality in the lower MCAi quartile (Q_1_) was 30.3 years (95% CI, 28.8–31.9) and 34.1 years (95% CI, 33.3–34.8) in the upper MCAi quartiles (Q_2-4_)

Kaplan–Meier survival curves (Fig. 2a) and log-rank test demonstrated a statistically significant shorter time until death for the abnormal ECG group (P < 0.001), and for those in the abnormal quartile (Q_1_) of the MCAi P < 0.001 (Fig. 2b). This was also observed for the other insulin sensitivity indices. Kaplan–Meier survival curves (Fig. 2c) and log-rank test for cardiovascular mortality demonstrated a statistically significant shorter time until death for individuals in the MCAi lower quartile (Q_1_) *P* < 0.001.

Median survival times of individuals in the lower quartile (Q_1_) of MCAi was 28 years (95% CI, 26.6–29.4) and 33 years (95% CI: 31.9–34.1) in the upper MCAi quartiles (Q_2-4_), Log-rank test: *p* < 0.001. Table 3 presents the results of the univariate and multivariate Cox regression analyses. Adjusting for age, sex, origin, BMI, blood pressure, cholesterol, smoking and glycemic state. Individuals in the lower quartile of MCAi showed a 20% greater all-cause mortality risk compared with the upper quartiles (95% CI: 1.02–1.3, *P* = 0.02). An increased risk for all-cause mortality was also observed in Q_4_-Ln HOMA-IR,Q_1_-QUICKI and Q_4_–fasting glucose as well, HR = 1.2 (95% CI, 1.04–1.4,* P* = 0.01), HR = 1.2 (95% CI, 1.04–1.4,* P* = 0.01) and HR = 1.3 (95% CI, 1.2–1.5, *P* < 0.001) respectively. Male sex, smoking status, diabetes morbidity, abnormal blood pressure, and obesity, were all found to significantly associate with all-cause mortality.

**Table 3:** Cox proportional hazards regression results (unadjusted and adjusted ^a^) for 31-year (median) all-cause and cardiovascular mortality

Characteristic	Reference category	All-cause mortality				Cardiovascular mortality			
		Univariate HR (95% CI)	Univariate *P*-value	Multivariate HR (95% CI)	Multivariate *P*-value	Univariate HR (95% CI)	Univariate *P*-value	Multivariate HR (95% CI)	Multivariate *P*-value
Age	1-year increment	1.1 (1.1–1.12)	< 0.001	1.1 (1.09–1.1)	< 0.001	1.2 (1.1–1.2)	< 0.001	1.1 (1.1–1.2)	< 0.001
Sex, Male	Female	1.4 (1.3–1.6)	< 0.001	1.2 (1.1–1.4)	0.001	1.5 (1.2–1.9)	< 0.001	1.4 (1.1–1.7)	0.006
Origin	Yemen								
Middle East		1.04 (0.9–1.2)	0.6	1.3 (1.1–1.5)	0.004	1.01 (0.8–1.3)	0.96	1.4 (1.1–1.9)	0.02
North Africa		0.96 (0.8–1.1)	0.6	1.1 (0.95–1.3)	0.3	1.02 (0.8–1.3)	0.9	1.2 (0.9–1.6)	0.1
Europe-America		1.2 (0.99–1.4)	0.08	1.2 (1.02–1.4)	0.06	1.1 (0.8–1.5)	0.5	1.04 (0.8–1.4)	0.8
Smoking status, Ever	Never	1.2 (1.03–1.3)	0.01	1.2 (1.03–1.3)	0.04	1.2 (1.02–1.5)	0.03	1.1 (0.9–1.4)	0. 3
Glycemic state	Normoglycemia								
Prediabetes		1.5 (1.3–1.7)	< 0.001	1.1 (0.95–1.2)	0.2	1.8 (1.4–2.2)	< 0.001	1.1 (0.9–1.4)	0.6
Diabetes		3.5 (2.9–4.1)	< 0.001	1.8 (1.5–2.1)	< 0.001	6.6 (4.9–8.8)	< 0.001	2.1 (1.5–2.9)	< 0.001
Systolic Blood Pressure	1-mmHg increment	1.7 (1.5–1.9)	< 0.001	1.01 (1.004–1.01)	< 0.001	1.03 (1.03–1.04)	< 0.001	1.01 (1.007–1.02)	< 0.001
BMI (${\mathrm{Kg}/\mathrm{m}}^{2}$) ^b^	Normal								
Overweight		1.3 (1.1–1.4)	< 0.001	1.1 (0.97–1.2)	0.1	1.3 (1.02–1.6)	0.03	1.1 (0.8–1.4)	0.6
Obese		1.6 (1.4–1.9)	< 0.001	1.3 (1.1–1.6)	0.001	1.99 (1.5–2.6)	< 0.001	1.4 (1.03–1.9)	0.03
Total cholesterol^c^ Borderline-high High	Normal	1.03 (0.9–1.2) 1.2 (1.0–1.4)	0.5 0.004	0.98 (0.8–1.1) 1.04 (0.9–1.2)	0.7 0.6	1.03 (0.8–1.3) 1.5 (1.2–1.9)	0.9 0.002	0.99 (1.3–1.8) 1.2 (0.9–1.5)	0.9 0.2
Ln MISI, Q_1_	Q_2-4_	1.4 (1.2–1.7)	< 0.001	1.03 (0.9–1.2)	0.2	1.7 (1.2–2.2)	0.01	0.9 (0.6–1.2)	0.1
Ln HOMA-IR, Q_4_	Q_1-3_	1.5 (1.3–1.7)	< 0.001	1.2 (1.04–1.4)	0.01	1.8 (1.4–2.2)	< 0.001	1.2 (0.95–1.5)	0.09
Ln HOMA-%B, Q_1_	Q_2-4_	1.3 (1.1–1.4)	< 0.001	0.96 (0.8–1.1)	0.7	1.4 (1.1–1.8)	0.002	0.9 (0.7–1.2)	0.7
QUICKI, Q_1_	Q_2-4_	1.5 (1.3–1.7)	< 0.001	1.2 (1.04–1.4)	0.01	1.8 (1.5–2.2	< 0.001	1.2 (0.9–1.5)	0.09
MCAi, Q_1_	Q_2-4_	1.3 (1.1–1.5)	< 0.001	1.2 (1.02–1.3)	0.03	1.6 (1.3–2.03)	< 0.001	1.4 (1.1–1.8)	0.01
MCAi	1-unit increment	0.8 (1.8–0.9)	< 0.001	0.9 (0.8–0.97)	0.06	0.7 (0.6–0.8)	< 0.001	0.8 (0.7–0.96)	0.002
Fasting Insulin, Q_4_	Q_1-3_	1.2 (1.1–1.4)	0.003	1.1 (0.95–1.2)	0.2	1.3 (1.05–1.6)	0.02	1.1 (0.9–1.4)	0.4
Fasting Glucose, Q_4_	Q_1-3_	1.9 (1.7–2.2)	< 0.001	1.3 (1.2–1.5)	< 0.001	2.6 (2.1–3.2)	< 0.001	1.5 (1.2–1.9)	0.001
Triglycerides	1- mg/dl increment	1.0 (1.0–1.001)	0.003	1.0 (1.0–1.001)	0.5	1.0 (1.0–1.0)	0.001	1.0 (0.99–1.0)	0.9

*MISI* Matsuda Insulin Sensitivity Index, *HOMA-IR* Homeostatic model assessment -Insulin resistance, *HOMA-%B* Homeostatic model assessment–percent beta cell function; *QUICKI* Quantitative Insulin Sensitivity Check Index; *MCAi* Mcauley index

^a^Multivariable models were adjusted to the MCAi and not the other insulin sensitivity indices; ^b^BMI categories: Normal < 25 kg/m^2^, Overweight, 25–29.9 kg/m^2^; Obese-BMI ≥ 30 kg/m^2^; ^c^Total cholesterol classification: Normal < 200 mg/dl, Borderline-high, 200–239 mg/dl, High ≥ 240 mg/dl

After adjusting for age, sex, origin, BMI, blood pressure, total cholesterol, smoking and glycemic state, a greater risk for cardiovascular mortality was observed for individuals in the lower quartile of MCAi, compared with upper quartiles (HR = 1.4, 95%CI: 1.1–1.8, *p* = 0.007).Similar findings were also observed for individuals in the upper quartile of fasting glucose (HR = 1.5, 95%CI: 1.2–1.9, *p* = 0.001). The remaining indices did not demonstrate a significant risk for cardiovascular mortality (Table 3).

Insulin resistance, expressed by the Q_1_ -MCAi significantly associated with all-cause mortality (adjusted HR = 1.2, 95% CI 1.1- 1.4) and with a borderline significance for cardiovascular mortality (adjusted HR = 1.3, 95% CI 0.99–1.7) in the non-diabetic cohort members (Additional file 1: Table S4c) whereas Q_4_-fasting glucose did not show a significant association with all-cause and cardiovascular mortality after the exclusion of diabetic individuals from the cohort (adjusted HR = 1.1, 95% CI 0.96–1.2) and (adjusted HR = 1.04, 95% CI 0.8–1.3) respectively.

## Discussion

The current study, performed on ethnically heterogeneous cohort of men and women, has shown a significant association between insulin resistance (IR), reflected by the MCAi and ischemic changes on ECG (Q-QS abnormality, ST elevation). Persistence of the association between the MCAi and ischemic changes on ECG, when excluding individuals with diabetes from the multivariable model, emphasize the association between IR and cardiovascular morbidity.

These findings are in line with other studies on insulin sensitivity indices, particularly abnormal MCAi values, and increased risk for CHD [22, 24, 31]. Effoe et al. [31] followed 3565 black men and women, free of diabetes mellitus and cardiovascular disease at baseline, for CAD incidence, over a median follow‐up of 8.4 years [31]. They showed a decreased risk for CAD with each SD increase in the MCAi (HR = 0.80; 95% CI: 0.67–0.96). Moreover, MCAi and HOMA‐IR were associated with CAD (HR = 0.71, 95% CI: 0.55–0.92 and HR = 1.33, 95% CI: 1.03–1.72, respectively), but not with stroke risk.

To point out, Q and QS Patterns and ST segment elevation are primarily associated with CAD [32, 33]. However, other etiologies should be considered, such as left ventricular hypertrophy, effect of medications (e.g. digitalis), or infiltrative diseases such as cardiac amyloidosis.

Sex, age, hypertension and obesity in the current study were also associated with an increased risk for the occurrence of ischemic changes, as expected, as they are all well-known cardiovascular risk factors.

Our findings demonstrate an additional risk of 20% for all-cause mortality and 40% for cardiovascular mortality in cohort members of the MCAi lower quartile compared with upper quartiles, independently of the presence of diabetes. Furthermore, the abnormal quartiles of Ln HOMA-IR and QUICKI were also associated with an additional 20% risk for all-cause mortality and for cardiovascular mortality, but reached statistical significance for all-cause mortality only.

All-cause mortality was mainly attributed to cardiovascular mortality (20.6%) as the primary cause of death in the cohort. The secondary cause of death was malignancy associated mortality (15.8%). In addition to the MCAi, male sex, age, origin (Middle Eastern), obesity, high blood pressure and diabetes, were also associated with a higher risk for cardiovascular mortality.

MCAi was the only ISI that showed a significant association with ischemic changes on ECG in addition to increased risk for all cause and cardiovascular mortality. Moreover, a significant association between MCAi lower quartile and all-cause mortality was observed even after excluding diabetic subjects from the cohort (as detailed below). This may be attributed to the inclusion of fasting triglycerides in the MCAi calculation. Fasting triglycerides reflects abnormal lipids metabolism as a direct and early outcome of insulin resistance [34–36] and perhaps increases the risk for coronary artery disease (CAD) and cardiovascular mortality [37, 38]. The direct association between increased triglycerides and cardiovascular morbidity and mortality remain controversial. However, several meta-analyses described an increased risk for CAD for individuals with abnormal triglycerides levels [37, 38]. A meta-analysis [37] from 2 prospective cohort studies on 44,237 Western middle-aged men and women, the Reykjavik study and the European Prospective Investigation of Cancer (EPIC)-Norfolk study, showed an increased risk for CHD after adjustment for cardiovascular risk factors (HR = 1.43, 95% CI: 1.23–1.65, and HR = 1.52, 95% CI: 1.24—1.89 for individuals in the top third of log-triglyceride in the Reykjavik and the Norfolk studies, respectively). Adjustment for cardiovascular risk factors substantially attenuated the above observed associations supporting the hypothesis that increased triglycerides reflect metabolic abnormalities such as diabetes and obesity that increases CVD incidence rather than a direct contribution [38]. Moreover, increased triglycerides further contribute to beta-cell dysfunction by a direct toxicity mechanism and enhances the insulin resistance state and therefore increases the risk for cardiovascular morbidity and mortality [35].

In line with other studies [22, 39], the present study further supports the use of MCAi as an accurate and early detection methods for insulin resistance compared with other ISI. Kim, T. J et al. [39] demonstrated that MCAi had the strongest correlation with insulin resistance, the highest area under the curve, specificity, positive predictive value and negative predictive value to distinguish individuals with metabolic syndrome from healthy subjects.

The study population mainly consisted of non-diabetic subjects and only 218 (11.9%) participants were diagnosed with diabetes at baseline. A sensitivity analysis excluding examinees with the diagnosis of diabetes, comprised of n = 1612 non diabetic individuals, and did not reveal a statistically significant association between ISIs and ECG findings. However, an increased risk for all-cause and cardiovascular mortality was observed (HR = 1.2, 95%CI: 1.1–1.4, and HR = 1.3, 95%CI: 0.99–1.7, respectively) for individuals in the MCAi lower quartile (Q_1_) compared to upper quartiles (Q_2-4_). Although fasting glucose upper quartile (Q_4_) was associated with an increased risk for all-cause and cardiovascular mortality, in the non-diabetic cohort the associations decreased in strength and did not reached statistical significant. In addition, fasting glucose models were not adjusted for glycemic state due to multi-collinearity.

In spite of the widespread use of ISI's in epidemiological studies, their clinical application is still uncommon and difficult to implement. Placzkowska et al. [40] described key challenges for ISIs implementation in clinical practice such as the lack of international standardization in insulin and glucose laboratory measurements and the absence of reference intervals and cut-off values according to the Clinical and Laboratory Standards Institute recommendations in the general population and in different age, sex, BMI and ethnicity subgroups.

Despite these limitations, our findings underscore the importance of the MCAi as a potentially sensitive biomarker in respect to other and more prevalent markers for metabolic abnormalities in the general population and specifically in non-diabetic individuals and their prognostic value, calling for further evaluation. Strengths and limitations.

Our findings should be interpreted under the following limitation: The oral glucose tolerance test (OGTT), was carried out using 100 gr of glucose ingestion instead of 75 gr as recommended by the American Diabetes Association [28], since at the time of the examination (prior to the recommendations, 1979–1982) no clear guidelines were present for this test. In addition, the use of 100gr of glucose instead of 75 gr, was reported to enhance the insulin response and insulin secretion [41], and to have a minimal effect on the glucose level and OGTT results 42).

Another limitation is the oversampling of Yemenites in the GOH cohort, which was done in order to provide statistical power to study this minority in relation to hypertension and diabetes incidence. In addition, the current analysis included a subsample of the target population with the full set of data (ECG, fasting glucose and insulin) with a higher proportion of males and of European American origin. While this may reduce the external validity of the study, the multivariable analysis was adjusted for sex and ethnicity.

In the current study, MISI mean glucose plasma levels was calculated using 0,60 and 120 min after OGTT and mean insulin plasma levels using 0, 30, 60 and 120 min after OGTT. However, the use of fewer measurements for the mean insulin and glucose calculation is acceptable in the literature [10, 11, 43]. In addition, only participants with the presence of every insulin and glucose measurement after OGTT were included for MISI calculation (n = 1071). A sensitivity analysis was performed including participants with existing data from every glucose and insulin measurements available (n = 1830) for the calculation of MISI with similar findings.

Despite these limitations, the study presents a number of key advantages: this is a cohort study with both men and women, representing the diverse population of the Israeli- Jewish population, with a prolonged follow up time of 40 years. Furthermore, all ECGs were interpreted by a single cardiologist, avoiding inter-observer variability, and blood tests were performed for research purposes only by a single lab which conformed to the highest standards. In addition, our analysis pertained to both a highly sensitive definition of any ECG abnormality, as well as to 11 specific major and minor ECG changes, defined according to the Minnesota instrument.

To conclude, our findings demonstrate an association between higher insulin resistance, presented by the lower quartile of the MCAi, and ischemic changes on ECG. MCAi lower quartile was associated with higher risk for approximately 40-year all-cause and cardiovascular mortality in an adult population regardless of the presence of diabetes, and may be consider as a simple and readily available biomarker for early cardiovascular signs and for greater mortality risk.

## Supplementary Information

**Additional file 1:** **Table S1.** Cohort baseline characteristics according to survival status and cause of death. **Table S2.** Classification and prevalence of ECG findings. **Table S3.** Unadjusted and adjusted logistic regression models for ECG findings. **Table S4.** **a** Insulin sensitivity indices quartiles distribution of 1830 men and women according to glycemic state. **b** Associations between insulin sensitivity indices and ECG findings: unadjusted and adjusted^a^ logistic regression results, excluding diabetic individuals (N=1612). **C** The association between insulin sensitivity indices and all-cause and cardiovascular mortality—Cox proportional hazard models, excluding diabetic individuals.

## Data Availability

The datasets used and/or analysed during the current study are available from the corresponding author on reasonable request.

## Abbreviations

HOMA-IR: Homeostatic model assessment -Insulin resistance
HOMA-%B: Homeostatic model assessment–percent beta cell function
MISI: Matsuda Insulin Sensitivity Index
QUICKI: Quantitative Insulin Sensitivity Check Index
MCAi: Mcauley index
ISI: Insulin sensitivity indices
OGTT: Oral glucose tolerance test
