# Supplementary material for: The association between insulin sensitivity indices, ECG findings and mortality: a 40-year cohort study
Source: Cardiovasc Diabetol. 2021 May 6;20:97. doi: 10.1186/s12933-021-01284-9 (PMC8103608; doi:10.1186/s12933-021-01284-9)
Supplement: Supplementary file 1 — Additional file 1: Table S1. Cohort baseline characteristics according to survival status and cause of death. Table S2. Classification and prevalence of ECG findings. Table S3. Unadjusted and adjusted logistic regression models for ECG findings. Table S4. a Insulin sensitivity indices quartiles distribution of 1830 men and women according to glycemic state. b Associations between insulin sensitivity indices and ECG findings: unadjusted and adjusteda logistic regression results, excluding diabetic individuals (N=1612). C The association between insulin sensitivity indices and all-cause and cardiovascular mortality—Cox proportional hazard models, excluding diabetic individuals. [file 12933_2021_1284_MOESM1_ESM.docx]

**Appendices:**

Table S-1: Cohort baseline characteristics according to survival status and cause of death

| **Vital status by end of follow-up*** | | |  |
| --- | --- | --- | --- |
| **Cardiovascular mortality** | **All-cause mortality** | **Alive** |  |
| n=377 | n=1,276 | n=554 | Characteristic |
| 56.3±7.1 | 54.7±7.4 | 45.8±5.8 | Age, years, mean ± SD |
| 102 (27.1)  69 (18.3)  79 (21.0)  127 (33.7) | 326 (25.5)  246 (19.3)  285 (22.3)  419 (32.8) | 148 (26.7)  90 (16.2)  116 (20.9)  200 (36.1) | Origin, n (%)  Middle East  North Africa  Yemen  Europe-America |
| 209 (55.4)  168 (44.6) | 698 (54.7)  578 (45.3) | 238 (43.0)  316 (57.0) | Sex, n (%)  Male  Female |
| 159 (42.2)  218 (57.8) | 525 (41.2)  750 (58.8) | 203 (36.6)  351 (63.4) | Smoking status ^a^, n (%)  Smoker  Non-Smoker |
| 109 (28.9)  186 (49.3)  82 (21.8) | 439 (34.4)  635 (49.8)  202 (15.8) | 302 (54.5)  236 (42.6)  16 (2.9) | Glycemic state, n (%)  Normoglycemia  Prediabetes  Diabetes |
| 142.9±27.3  87.6±15.7 | 137.1± 26.7  85.5±15 | 121.8±22.7  80.6±14.1 | Blood Pressure, mmHg, mean ± SD  Systolic  Diastolic |
| 25.8 [5.4]  157 (41.6)  147 (39)  73 (19.4) | 26.1 [5.3]  499 (39.1)  543 (42.6)  234 (18.3) | 24.5 [4.1]  308 (55.6)  195 (35.2)  51 (9.2) | BMI (${Kg/M}^{2}$)^b^ , median [IQR]  Normal, n (%)  Overweight, n (%)  Obese, n (%) |
| 115.2±42.7  246 (65.3)  131 (34.7) | 109.2 ±33.7  871 (68.3)  405 (31.7) | 97.1 ±14.4  477 (86.1)  77 (13.9) | Fasting glucose, mg/dl, mean ± SD  Q1-3, n (%)  Q4, n (%) |
| 17.8±13.2  273 (72.4)  104 (27.6) | 16.9 ±12.1  931 (73)  345 (27) | 15.2 ±11.2  434 (78.3)  120 (21.7) | Fasting insulin, mU/L, mean ± SD  Q1-3, n (%)  Q4 |
| 232.3±57.4  102 (27.1)  107 (28.4)  168 (44.6) | 224 ±55.2  394 (30.9)  389 (30.5)  493 (38.6) | 214.3 ±52.0  201 (36.3)  184 (33.2)  169 (30.5) | Total cholesterol ^c^, mg/dl, mean ± SD  Normal, n (%)  Borderline high, n (%)  High, n (%) |
| 120 [87.5] | 115 [75] | 100 [71.3] | Triglycerides, mg/dl, median [IQR] |
| 3.0 [2.0]  1.1± 0.5  60 (30)  140 (70) | 3.3 [2.3]  1. 2 ± 0.6  202 (28.5)  508 (71.5) | 3.9 [2.4]  1.3 ± 0.5  65 (18.1)  295 (81.9) | MISI, median [IQR]  Ln MISI  Q1 n (%)  Q_2-4_, n(%) |
| 3.8 [2.8]  1.4 ± 0.7  261 (69.2)  116 (30.8) | 3.6 [2.8]  1.3 ± 0.7  915 (71.7)  361 (28.3) | 3.0 [2.0]  1.1 ± 0.6  458 (82.7)  96 (17.3) | HOMA-IR, median [IQR]  Ln HOMA-IR,  Q_1-3_, n (%)  Q_4_, n (%) |
| 127.7 [98.1]  4.8 ± 0.7  105 (27.9)  271 (72.1) | 130.4 [98.9]  4.9 ± 0.7  342 (26.9)  930 (73.1) | 144.0 [106.9]  4.9 ± 0.6  114 (20.6)  440 (79.4) | HOMA-%B, median [IQR]  Ln HOMA-%B,  Q_1_, n (%)  Q_2-4_, n (%) |
| 0.3 ± 0.03  116 (30.8)  261 (69.2) | 0.3 ± 0.1  361 (28.3)  915 (71.7) | 0.3 ± 0.1  96 (17.3)  458 (82.7) | QUICKI, mean ± SD  Q_1_, n (%)  Q_2-4_, n (%) |
| 3.7 ± 0.9  115 (31.5)  250 (68.5) | 3.8 ± 0.9  336 (27)  909 (73) | 4.0 ± 0.6  112 (20.4)  438 (79.6) | MCAi, mean ± SD  Q_1_, n (%)  Q_2-4_, n (%) |

MISI, Matsuda Insulin Sensitivity Index; HOMA-IR, Homeostatic model assessment -Insulin resistance; HOMA-%B, Homeostatic model assessment – percent beta cell function; QUICKI, Quantitative Insulin Sensitivity Check Index; MCAi, Mcauley index.

^a^ Smoking status classification: Smoker-current or past smoker. Nonsmoker-never smoked; ^b^ BMI classification: Normal < 25 kg/M^2^; Overweight- 25 – 29.9; Obese- BMI ≥ 30 kg/M^2^; ^d^ Total cholesterol classification: Normal < 200 mg/dl; Borderline high 200–239 mg/dl; High ≥ 240 mg/dl.

Table S-2: Classification and prevalence of ECG findings

| **ECG finding category** | **Description** | **ECG finding category** | **Description** |
| --- | --- | --- | --- |
| Left Axis Deviation + Nonspecific T wave changes (n=710) |  | Left axis deviation (n=140) |  |
| Nonspecific T (NST) wave changes (n=605) | Terminal T negativity  Peak T wave  Nonspecific T (NST) changes –2,3, aVf  Prominent turn counter clock  Prominent turn clock  NST 1, aVL,V5-6  NST V1-4 | Ischemic changes (n=128) | Q and QS Patterns:  OLD MI  OLD MI suspect  OLD MI suspect- diaphragm  OLD MI suspect- anterior (ant) septal  OLD MI suspect- ant lateral  OLD MI suspect- ant extended  OLD MI suspect- Lateral  OLD MI suspect- High Lateral  OLD MI suspect- True post  OLD MI suspect- sub endocardial ischemia  Coronary insufficiency  ST-elevation:  MI  MI Diaphragm  MI anterior  MI Lateral  MI posterior  Persistence STE post MI |
| Miscellaneous items (n=494) | Low voltage  P mitrale  P pulmonale  Other  Drug effect  Long QT interval (LQT)  High take off | Arrhythmia (n=122) | Atrial fibrillation  Atrial flutter  Atrial Tachycardia  Wandering pacemaker  Junctional pacemaker  Few SVPB  Many SVPB  Wandering VPB  Many VPB |
| ST Junction (J) and segment depression (n=492) | Diastolic overload  Left Ventricular strain | Atrioventricular conduction defect (AVCD) (n=48) | First degree AV Block  Short PR  Wolf Parkinsons white syndrome (WPW) |
| Nonspecific ST (NSST) changes (n=393) | Nonspecific ST changes –2,3, aVf  NSST -1, aVL,V5-6  NSST V1-4 | Right axis deviation (n=24) |  |
| Ventricular conduction defect (VCD) (n=332) | Complete left bundle branch block (CLBBB)  Complete right bundle branch block (CRBBB)  Intermittent RBBB  Intermittent LBBB  Intra ventricular conduction delay (IVCD)  Incomplete RBBB  Incomplete LBBB  V1 RsR  Slow progression of R V1-V3 |  |  |

| **Arrhythmias**  n**=**122 | **Ventricular Conduction Defect**  n**=**332 | **A-V Conduction Defect**  n**=**48 | **Left Axis Deviation**  n=140 | **Right axis Deviation**  n=24 | **Any ECG abnormality**  n=914 | ISI (highest vs lower IR quartile) |
| --- | --- | --- | --- | --- | --- | --- |
| 1.4 (0.9-2.1) | 1.2 (0.8-1.6) | 2.1 (1.1-3.8)* | 1.4 (0.9-2.1) | 1.1 (0.4-2.9) | 1.4 (1.2-1.8)*** | QUICKI (Q_1_ vs Q_2-4_) Cr. |
| 1.03 (0.6-1.7) | 1.1 (0.8-1.6) | 1.7 (0.8-1.5) | 1.1 (0.7-1.8) | 1.4 (0.4-4.4) | 1.2 (0.9-13.6) | Adjusted |
| 2.02 (1.2-3.6)**  1.8 (0.9-3.7) | 1.6 (1.04-2.3)*  1.5 (0.9-2.4) | 1.5 (0.6-3.5)  1.6 (0.6-4.3) | 1.6 (0.9-2.8)  1.2 (0.6-2.5) | 2.2 (0.8-6.3)  3.2 (0.8-12.8) | 1.6 (1.2-2.1)***  1.3 (0.99-1.8)* | Ln MISI (Q_1_ vs Q_2-4_) Cr.  Adjusted |
| 1.4 (0.9-2.1) | 1.2 (0.8-1.6) | 2.1 (1.1-3.8)* | 1.4 (0.9-2.1) | 1.1 (0.4-2.9) | 1.4 (1.2-1.8)** | Ln HOMA-IR (Q_4_ vs Q_1-3_) Cr. |
| 1.03 (0.6-1.7) | 1.1 (0.8-1.6) | 1.7 (0.8-3.5) | 1.1 (0.7-1.8) | 1.4 (0.4-4.4) | 1.2 (0.95-1.5) | Adjusted |
| 1.7 (1.1-2.6)* | 1.5 (1.1-2.04)** | 1.3 (0.6-2.5) | 1.5 (0.99-2.3) | 0.8 (0.3-2.2) | 1.2 (0.9-1.5) | Ln HOMA-%B (Q_1_ vs Q_2-4_) Cr. |
| 1.3 (0.8-2.1) | 1.2 (0.8-1.7) | 0.9 (0.4-2.04) | 0.95 (0.6-1.6) | 0.7 (0.2-2.3) | 1.03 (0.8-1.3) | Adjusted |
| 0.9 (0.6-1.5)  0.8 (0.5-1.3) | 0.9 (0.7-1.2)  0.8 (0.6-1.2) | 1.4 (0.7-2.6)  1.001 (0.5-2.05) | 0.9 (0.6-1.4)  0.8 (0.5-1.3) | 0.3 (0.06-1.2)  0.2 (0.05-1.1) | 1.2 (0.99-1.5)  1.1 (0.8-1.4) | MCAi (Q_1_ vs Q_2-4_) Cr.  Adjusted |
| 0.95 (0.6-1.5) | 0.9 (0.7-1.3) | 1.6 (0.8-2.99) | 0.9 (0.6-1.4) | 0.98 (0.4-2.5) | 1.2 (0.96-1.5) | Fasting Insulin (Q_4_ vs Q_1-3_) Cr. |
| 0.8 (0.5-1.3) | 0.9 (0.6-1.3) | 1.3 (0.7-2.5) | 0.8 (0.5-1.3) | 0.98 (0.4-2.7) | 1.1 (0.8-1.3) | Adjusted |
| 1.4 (0.9-2.2) | 1.4 (1.02-1.9)* | 1.5 (0.8-2.8) | 1.99 (1.3-2.9)** | 0.7 (0.2-1.9) | 1.5 (1.2-1.9)*** | Fasting glucose (Q_4_ vs Q_1-3_) Cr. |
| 1.01 (0.6-1.6) | 0.96 (0.7-1.3) | 1.2 (0.6-2.3) | 1.3 (0.8-1.99) | 0.5 (0.1-2.8) | 1.03 (0.8-1.4) | Adjusted |

Table S-3: Unadjusted and adjusted logistic regression models for ECG findings

ISIs, Insulin Sensitivity Indices; IR, Insulin resistance; QUICKI, Quantitative Insulin Sensitivity Check Index; Cr., Crude ; MISI, Matsuda Insulin Sensitivity Index; HOMA-IR, Homeostatic model assessment -Insulin resistance; HOMA-%B, Homeostatic model assessment – percent beta cell function; MCAi, Mcauley index; *P<0.05, **P<0.01, ***P<0.001; Adjusted for: Age, Sex, Origin, BMI category, Total Cholesterol, Smoking, Glycemic state, Blood Pressure category.

Table S-3: Unadjusted and adjusted ^a^ logistic regression models for ECG findings (continue)

| **Lt Axis Dev & Nonspecific T-WC ^b^**  n**=**710 | **Ischemic changes**  n**=**128 | **Nonspecific ST changes**  n**=**393 | **Nonspecific T wave changes**  n**=**605 | **Miscellaneous Items**  n=494 | **ST Junction and Segment Depression**  n=492 | ISI (highest vs lower IR quartile) |
| --- | --- | --- | --- | --- | --- | --- |
| 1.3 (1.04-1.7)* | 1.7 (1.1-2.6)** | 1.3 (0.9-1.7) | 1.3 (1.02-1.7)* | 1.1 (0.8-1.5) | 1.5 (1.2-1.99)** | QUICKI (Q_1_ vs Q_2-4_) Cr. |
| 1.1 (0.8-1.5) | 1.3 (0.8-2.2) | 1.1 (0.8-1.6) | 1.1 (0.8-1.5) | 0.99 (0.7-1.4) | 1.2 (0.9-1.7) | Adjusted |
| 1.9 (1.1-3.4)*  1.8 (0.9-3.6) | 1.4 (0.9-2.1)  1.3 (0.8-2.1) | 1.2 (0.5-2.9) 1.2 (0.4-3.2) | 1.6 (0.9-2.8) 1.2 (0.6-2.4) | 1.6 (0.5-4.8) 1.7 (0.4-6.3) | 1.5 (1.1-1.99)  1.3 (0.95-1.5) | Ln MISI (Q_1_ vs Q_2-4_) Cr.  Adjusted |
| 1.3 (1.04-1.7)* | 1.7 (1.1-2.6)** | 1.3 (0.9-1.7) | 1.3 (1.02-1.7)* | 1.1 (0.8-1.5) | 1.5 (1.2-1.99)** | Ln HOMA-IR (Q_4_ vs Q_1-3_) Cr. |
| 1.1 (0.8-1.5) | 1.3 (0.8-2.2) | 1.1 (0.8-1.6) | 1.1 (0.8-1.5) | 0.99 (0.7-1.4) | 1.2 (0.9-1.7) | Adjusted |
| 1.4 (1.1-1.8)** | 1.1 (0.7-1.8) | 1.4 (1.02-1.9)* | 1.3 (1.02-1.8)* | 1.3 (0.99-1.7) | 1.2 (0.9-1.6) | Ln HOMA-%B (Q_1_ vs Q_2-4_) Cr. |
| 1.2 (0.9-1.6) | 0.7 (0.4-1.1) | 1.2 (0.8-1.6) | 1.2 (0.9-1.6) | 1.1 (0.8-1.6) | 1.02 (0.7-1.4) | Adjusted |
| 1.1 (0.9-1.4)  0.98 (0.7-1.3) | 1.9 (1.3-2.9)**  1.7 (1.02-2.8)* | 1.04 (0.8-1.4)  0.98 (0.7-1.4) | 1.2 (0.9-1.5)  1.02 (0.8-1.4) | 0.99 (0.8-1.3)  0.9 (0.6-1.2) | 1.2 (0.9-1.6)  1.2 (0.8-1.6) | MCAi (Q_1_ vs Q_2-4_) Cr.  Adjusted |
| 1.1 (0.8-1.4) | 1.2 (0.8-1.9) | 1.1 (0.8-1.5) | 1.1 (0.9-1.4) | 0.9 (0.7-1.2) | 1.2 (0.9-1.6) | Fasting Insulin (Q_4_ vs Q_1-3_) Cr. |
| 0.9 (0.7-1.2) | 1.005 (0.6-1.6) | 1.04 (0.8-1.4) | 0.96 (0.7-1.3) | 0.9 (0.6-1.2) | 1.1 (0.8-1.4) | Adjusted |
| 1.4 (1.1-1.9)**  1.03 (0.7-1.5) | 2.1 (1.4-3.1)***  1.1 (0.7-1.8) | 1.3 (0.98-1.8)  1.1 (0.8-1.6) | 1.3 (1.02-1.7)*  1.02 (0.8-1.4) | 1.2 (0.9-1.6)  0.9 (0.7-1.2) | 1.5 (1.1-1.9)**  1.2 (0.9-1.6) | Fasting glucose (Q_4_ vs Q_1-3_) Cr.  Adjusted |

ISIs, Insulin Sensitivity Indices; IR, Insulin resistance; QUICKI, Quantitative Insulin Sensitivity Check Index; Cr., Crude ; MISI, Matsuda Insulin Sensitivity Index; HOMA-IR, Homeostatic model assessment -Insulin resistance; HOMA-%B, Homeostatic model assessment – percent beta cell function; MCAi, Mcauley index; ^a^ Adjusted for: Age, Sex, Origin, BMI category, Total Cholesterol, Smoking, Glycemic state, Systolic Blood Pressure; ^b^Left Axis Deviation & Nonspecific T-wave changes ; *P<0.05, **P<0.01, ***P<0.001.

Table S-4a: Insulin sensitivity indices quartiles distribution of 1,830 men and women according to glycemic state.

| ISI (highest vs lower IR quartile) | **Normoglycemia**  n=741 | **Prediabetes**  n=871 | **Diabetes**  n=218 | **P-value** |
| --- | --- | --- | --- | --- |
| QUICKI, n (%)  Q_1_  Q_2-4_ | 79 (10.7)  662 (89.3) | 238 (27.3)  633 (72.7) | 140 (64.2)  78 (35.8) | *** |
| MCAi, n (%)  Q_1_  Q_2-4_ | 117 (16.0)  614 (84.0) | 232 (27.2)  620 (72.8) | 99 (46.7)  113 (53.3) | *** |
| Ln MISI, n (%)  Q_1_  Q_2-4_ | 51 (11.8)  382 (88.2) | 161 (29.2)  390 (70.8) | 55 (64.0)  31 (36.0) | *** |
| Ln HOMA-IR, n (%)  Q_1-3_  Q_4_ | 662 (89.3)  79 (10.7) | 633 (72.7)  238 (27.3) | 78 (35.8)  140 (64.2) | *** |
| Ln HOMA-%B, n (%)  Q_1_  Q_2-4_ | 81 (11.0)  658 (89.0) | 242 (27.8)  628 (72.2) | 133 (61.3)  84 (38.7) | *** |

ISI, Insulin Sensitivity Index; IR, Insulin resistance; QUICKI, Quantitative Insulin Sensitivity Check Index; Q_1_, First quartile; Q_2-4_, 2^nd^ to 4^th^ quartiles; ***P<0.001; MCAi, Mcauley index; MISI, Matsuda Insulin Sensitivity Index; HOMA-IR, Homeostatic model assessment -Insulin resistance; HOMA-%B, Homeostatic model assessment – percent beta cell function;. Table S-4b: Associations between insulin sensitivity indices and ECG findings: unadjusted and adjusted^a^ logistic regression results, excluding diabetic individuals (N=1612)

| **Arrhythmias** | **Ventricular Conduction Defect** | **A-V Conduction Defect** | **Left Axis Deviation** | **Right axis Deviation** | **Any ECG abnormality** | ISI (highest vs lower IR quartiles) |
| --- | --- | --- | --- | --- | --- | --- |
| n**=**100 | n**=**285 | n**=**39 | n=116 | n=21 | n=784 |  |
| 1.2 (0.8-2.0) | 1.1 (0.8-1.6) | 1.5 (0.7-3.0) | 1.2 (0.8-1.9) | 1.3 (0.5-3.5) | 1.3 (1.0-1.6)* | QUICKI (Q_1_ vs Q_2-4_) Cr. |
| 1.1 (0.6-1.8) | 1.1 (0.8-1.6) | 1.2 (0.5-2.5) | 1.01 (0.6-1.7) | 1.6 (0.6-4.6) | 1.1 (0.9-1.6) | Adjusted |
| 1.5 (0.7-3.1) | 1.7 (1.1-2.8)* | 1.2 (0.4-3.6) | 1.7 (0.9-2.9) | 1.9 (0.6-5.8) | 1.4 (1.1-1.9)** | Ln MISI (Q_1_ vs Q_2-4_) Cr. |
| 1.4 (0.7-2.9) | 1.2 (0.8-2.0) | 1.2 (0.4-3.3) | 1.4 (0.7-2.9) | 2.8 (0.7-11.0) | 1.2 (0.9-1.7) | Adjusted |
| 1.3 (0.8-2.1) | 1.1 (0.8-1.6) | 1.5 (0.7-3.0) | 1.2 (0.8-1.9) | 1.3 (0.5-3.5) | 1.3 (1.0-1.6)* | Ln HOMA-IR (Q_4_ vs Q_1-3_) Cr. |
| 1.1 (0.6-1.9) | 1.1 (0.8-1.6) | 1.2 (0.5-2.5) | 1.01 (0.6-1.7) | 1.6 (0.6-4.6) | 1.2 (0.9-1.5) | Adjusted |
| 1.2 (0.7-1.9) | 1.5 (1.1-2.1)* | 1.1 (0.5-2.4) | 1.3 (0.8-2.0) | 0.9 (0.3-2.6) | 1.2 (0.9-1.5) | Ln HOMA-%B (Q_1_ vs Q_2-4_ ) Cr. |
| 1.2 (0.7-2.1) | 1.3 (0.9-1.8) | 1.1 (0.5-2.4) | 1.0 (0.6-1.8) | 0.96 (0.3-3.1) | 1.1 (0.9-1.4) | Adjusted |
| 1.0 (0.6-1.7)  0.8 (0.5-1.5) | 0.9 (0.6-1.2)  0.8 (0.6-1.2) | 1.1 (0.5-2.2)  0.7 (0.3-1.7) | 0.98 (0.6-1.4)  0.8 (0.5-1.5) | 0.5 (0.1-1.8)  0.5 (0.1-1.7) | 1.1 (0.9-1.4)  0.99 (0.8-1.3) | MCAi (Q_1_ vs Q_2-4_ ) Cr.  Adjusted |

ISI, Insulin Sensitivity Indices; IR, Insulin resistance; QUICKI, Quantitative Insulin Sensitivity Check Index; Cr., Crude; MISI, Matsuda Insulin Sensitivity Index; HOMA-IR, Homeostatic model assessment -Insulin resistance; HOMA-%B, Homeostatic model assessment – percent beta cell function; MCAi, Mcauley index; ^a^ Adjusted for: Age, Sex, Origin, BMI category, Total Cholesterol, Smoking, Glycemic state, Systolic Blood Pressure; *P<0.05, **P<0.01.

Table S-4b: Associations between ISIs and ECG findings: unadjusted and adjusted^a^ logistic regression results, excluding diabetic individuals (N=1612) (continue).

| **Lt Axis Dev & Nonspecific T-WC^b^** | **Ischemic changes** | **Nonspecific ST changes** | **Nonspecific T wave changes** | **Miscellaneous Items** | **ST Junction & Segment Depression** | ISI (highest vs lower IR quartiles) |
| --- | --- | --- | --- | --- | --- | --- |
| n**=**608 | n**=**105 | n**=**339 | n**=**522 | n=424 | n=421 |  |
| 1.3 (0.9-1.6) | 1.6 (1.0-2.6)* | 1.2 (0.9-1.7) | 1.3 (0.9-1.7) | 1.0 (0.8-1.4) | 1.4 (1.0-1.8)* | QUICKI (Q1 vs Q2-4)- Cr. |
| 1.1 (0.8-1.5) | 1.5 (0.8-2.5) | 1.2 (0.8-1.7) | 1.1 (0.8-1.5) | 0.99 (0.7-1.4) | 1.2 (0.9-1.6) | Adjusted |
| 1.4 (0.9-1.9) | 2.3 (1.2-4.5)* | 1.1 (0.7-1.7) | 1.5 (1.01-2.1)* | 1.3 (0.8-2.0) | 1.6 (1.05-2.6)* | Ln MISI- (Q1 vs Q2-4) Cr. |
| 1.1 (0.7-1.6) | 1.5 (0.7-3.0) | 1.0 (0.6-1.6) | 1.1 (0.7-1.6) | 0.9 (0.6-1.5) | 1.3 (0.9-2.0) | Adjusted |
| 1.3 (0.9-1.7) | 1.7 (1.1-2.6)* | 1.3 (0.9-1.7) | 1.3 (0.9-1.7) | 1.0 (0.8-1.4) | 1.4 (1.0-1.8)* | Ln HOMA-IR, (Q_4_ vs Q_1-3_), Cr. |
| 1.1 (0.8-1.5) | 1.5 (0.9-2.5) | 1.2 (0.8-1.7) | 1.1 (0.8-1.5) | 0.98 (0.7-1.4) | 1.2 (0.9-1.6) | Adjusted |
| 1.3 (0.99-1.7)* | 1.1 (0.7-1.8) | 1.4 (1.03-1.9)* | 1.3 (0.9-1.7) | 1.3 (0.99-1.8) | 1.2 (0.9-1.7) | Ln HOMA-%B (Q_1_ vs Q_2-4_) Cr. |
| 1.3 (0.9-1.7) | 0.8 (0.4-1.4) | 1.3 (0.9-1.9 | 1.3 (0.9-1.8) | 1.3 (0.9-1.8) | 1. 2 (0.8-1.6) | Adjusted |
| 1.2 (0.9-1.6)  1.1 (0.8-1.5) | 1.9 (1.2-2.9)**  1.6 (0.9-2.7) | 1.14 (0.8-1.5)  0.99 (0.7-1.4) | 1.3 (0.9-1.7)  1. 2 (0.9-1.6) | 0.95 (0.7-1.3)  0.8 (0.6-1.1) | 1.3 (0.9-1.8)  1.2 (0.9-1.7) | MCAi–Q_1_ vs Q_2-4 ,_ Cr.  Adjusted |

ISI, Insulin Sensitivity Indices; IR, Insulin resistance; QUICKI, Quantitative Insulin Sensitivity Check Index; Cr., Crude; MISI, Matsuda Insulin Sensitivity Index; HOMA-IR, Homeostatic model assessment -Insulin resistance; HOMA-%B, Homeostatic model assessment – percent beta cell function; MCAi, Mcauley index.

^a^ Adjusted for: Age, Sex, Origin, BMI category, Total Cholesterol, Smoking, Glycemic state, Systolic Blood Pressure; ^b^Left Axis Deviation & Nonspecific T-wave changes; *P<0.05.

Table S-4c: The association between insulin sensitivity indices and all-cause and cardiovascular mortality – Cox proportional hazard models, excluding diabetic individuals.

| **Cardiovascular mortality**  **HR (95%CI)** | | **All-cause mortality**  **HR (95%CI)** | | ISI (highest vs lower IR quartiles) |
| --- | --- | --- | --- | --- |
| Adjusted ^a^ | Unadjusted | Adjusted ^a^ | Unadjusted |  |
| 1.1 (0.8-1.4) | 1.2 (0.9-1.6) | 1.1 (0.9-1.3) | 1.2 (1.01-1.3)* | QUICKI (Q_1_ vs Q_2-4)_ |
| 1.1 (0.8-1.7) | 1.4 (1.01-1.9) | 1.1 (0.9-1.3) | 1.3 (1.1-1.6)* | Ln MISI (Q_1_ vs Q_2-4_) |
| 1.1 (0.8-1.4) | 1.2 (0.9-1.6) | 1.1 (0.9-1.3) | 1.2 (1.01-1.3)* | Ln HOMA-IR (Q_4_ vs Q_1-3_) |
| 0.99 (0.7-1.3) | 0.97 (0.7-1.3) | 1.0 (0.9-1.2) | 1.1 (0.9-1.2) | Ln HOMA-%B, (Q_1_ vs Q_2-4_) |
| 1.3 (0.99-1.7) | 1.3 (1.02-1.7)* | 1.2 (1.1-1.4)** | 1.2 (1.1-1.4) | MCAi, (Q_1_ vs Q_2-4_ ) |

ISIs, Insulin Sensitivity Indices; IR, Insulin resistance; QUICKI, Quantitative Insulin Sensitivity Check Index; MISI, Matsuda Insulin Sensitivity Index; HOMA-IR, Homeostatic model assessment -Insulin resistance; HOMA-%B, Homeostatic model assessment – percent beta cell function; MCAi, Mcauley index.

^a^ Adjusted for: Age, Sex, Origin, BMI category, Total Cholesterol, Smoking, Glycemic state, Systolic Blood Pressure; *P<0.05, **P<0.01.
